# Supplementary material for: The conserved HIV-1 spacer peptide 2 triggers matrix lattice maturation
Source: Nature. 2025 Feb 26;640(8057):258–64. doi: 10.1038/s41586-025-08624-9 (PMC11964938; doi:10.1038/s41586-025-08624-9)
Supplement: Supplementary file 1 — Gel source data. Uncropped images of western blots shown in Extended Data Fig. 2. [file 41586_2025_8624_MOESM1_ESM.pdf]

nature portfolio

https://doi.org/10.1038/s41586-025-08624-9

Supplementary information

The conserved HIV-1 spacer peptide 2 triggers matrix lattice maturation

In the format provided by the authors and unedited

Supplementary Figure 1

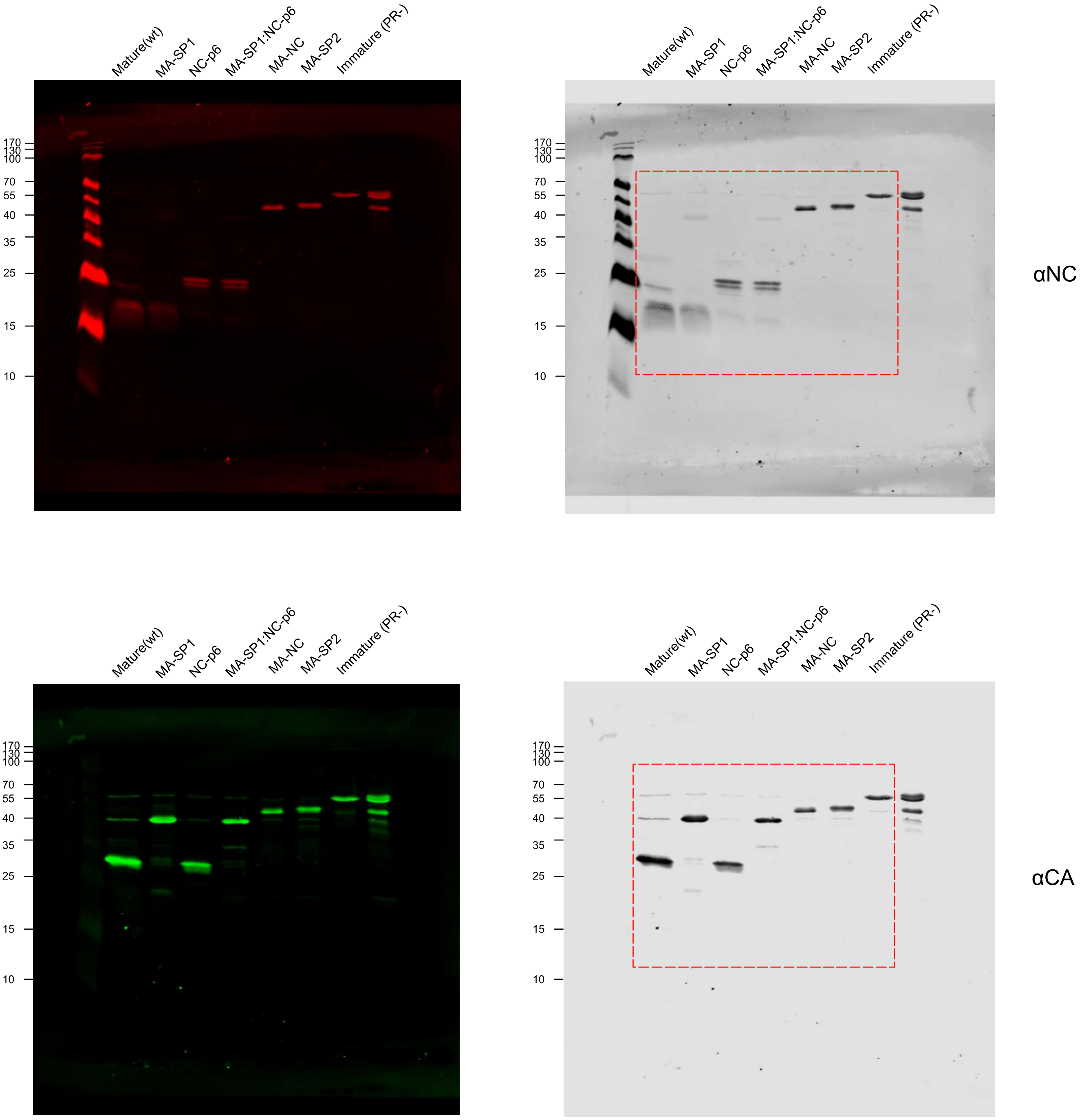

Uncropped western blots from extended data figure 2a.
