# Supplementary material for: The conserved HIV-1 spacer peptide 2 triggers matrix lattice maturation
Source: Nature. 2025 Feb 26;640(8057):258–64. doi: 10.1038/s41586-025-08624-9 (PMC11964938; doi:10.1038/s41586-025-08624-9)
Supplement: Supplementary file 2 — Reporting Summary [file 41586_2025_8624_MOESM2_ESM.pdf]

nature portfolio

Corresponding author(s): John Briggs

Last updated by author(s): Dec 17, 2024

Reporting Summary

Nature Portfolio wishes to improve the reproducibility of the work that we publish. This form provides structure for consistency and transparency in reporting. For further information on Nature Portfolio policies, see our Editorial Policies and the Editorial Policy Checklist.

Statistics

For all statistical analyses, confirm that the following items are present in the figure legend, table legend, main text, or Methods section.

n/a	Confirmed	
☐	☒	The exact sample size (*n*) for each experimental group/condition, given as a discrete number and unit of measurement
☐	☒	A statement on whether measurements were taken from distinct samples or whether the same sample was measured repeatedly
☐	☒	The statistical test(s) used AND whether they are one- or two-sided<br>*Only common tests should be described solely by name; describe more complex techniques in the Methods section.*
☒	☐	A description of all covariates tested
☒	☐	A description of any assumptions or corrections, such as tests of normality and adjustment for multiple comparisons
☐	☒	A full description of the statistical parameters including central tendency (e.g. means) or other basic estimates (e.g. regression coefficient) AND variation (e.g. standard deviation) or associated estimates of uncertainty (e.g. confidence intervals)
☐	☒	For null hypothesis testing, the test statistic (e.g. F, t, r) with confidence intervals, effect sizes, degrees of freedom and P value noted<br>*Give P values as exact values whenever suitable.*
☒	☐	For Bayesian analysis, information on the choice of priors and Markov chain Monte Carlo settings
☒	☐	For hierarchical and complex designs, identification of the appropriate level for tests and full reporting of outcomes
☒	☐	Estimates of effect sizes (e.g. Cohen's d, Pearson's r), indicating how they were calculated

Our web collection on statistics for biologists contains articles on many of the points above.

Software and code

Policy information about availability of computer code

Data collection	Cryo-electron microscopy data were collected using the ThermoFisher EPU software versions 3.3 - 3.8.
Data analysis	All software used for data analysis is widely available.<br>Cryo-electron microscopy data were analyzed using the following software: crYOLO v1.7.6; Relion 4.0 and 5.0; cryoSPARC v3.3 and 4.4; CTFfind4; MotionCorr2.<br>Model building and refinement of the structural model was perfomed using UCSF Chimera 1.15; ChimeraX 1.3; ModelAngelo v1.0; Phenix v1.21 and ISOLDE 1.3.<br>Fourier analysis of the 2D class averages was performed in MATLAB v2022a (MathWorks).<br>Data were visualized using ChimeraX; Fiji/imageJ v1.64f; and MATLAB v2022a.<br>Automated lipid fitting was performed by RosettaEmerald (in Rosetta 2023.45).<br>Fusion data was processed using Mathematica and Excel.

For manuscripts utilizing custom algorithms or software that are central to the research but not yet described in published literature, software must be made available to editors and reviewers. We strongly encourage code deposition in a community repository (e.g. GitHub). See the Nature Portfolio guidelines for submitting code & software for further information.

Data

Policy information about availability of data

All manuscripts must include a data availability statement. This statement should provide the following information, where applicable:

- Accession codes, unique identifiers, or web links for publicly available datasets
- A description of any restrictions on data availability
- For clinical datasets or third party data, please ensure that the statement adheres to our policy

Structures determined by electron microscopy are deposited in the Electron Microscopy Data Bank under accession codes EMD-52229, EMD-51769, EMD-52221 and EMD-52222. The molecular model of the MA-SP1 MA lattice is deposited in the Protein Data Bank under accession code PDB:9H1P, for which PDB:2H3I was used as a starting model.

Research involving human participants, their data, or biological material

Policy information about studies with human participants or human data. See also policy information about sex, gender (identity/presentation), and sexual orientation and race, ethnicity and racism.

Reporting on sex and gender	NA
Reporting on race, ethnicity, or other socially relevant groupings	NA
Population characteristics	NA
Recruitment	NA
Ethics oversight	NA

Note that full information on the approval of the study protocol must also be provided in the manuscript.

Field-specific reporting

Please select the one below that is the best fit for your research. If you are not sure, read the appropriate sections before making your selection.

☒ Life sciences ☐ Behavioural & social sciences ☐ Ecological, evolutionary & environmental sciences

For a reference copy of the document with all sections, see nature.com/documents/nr-reporting-summary-flat.pdf

Life sciences study design

All studies must disclose on these points even when the disclosure is negative.

Sample size	Cryo-EM of HIV-1 cleavage mutants particles: at least two grids were prepared independently for each mutant. Thousands of HIV-1 particles were imaged and used for averaging and reconstruction. For further details see methods. Sample size was determined by the availability of microscope time for imaging, and because the sample size was sufficient to determine the structures and lattice paramenters.<br>2D crystallography: the number of grids imaged, number of grid squares imaged per grid and images per grid square were chosen to representatively sample the whole cryo-EM grid while having sufficient data to obtain good quality 2D classes.<br>Virus fusion experiment: Sample size was standard three biological replicates with three technical replicate per biological replicate. Each biological replicate represents one aliquot from a bulk preparation of viral particles.
Data exclusions	2D crystallography and cryo-EM of cleavage mutants: Images were subjected to image classification procedures resulting in exclusion of some images. Image classification is a standard part of cryo-electron microscopy pipelines and are described in detail in the methods and Extended Data figures.<br>Virus fusion experiment: Data were not exluded.
Replication	cryo-EM data: At least two grids were prepared and imaged from each HIV-1 cleavage mutant sample. HIV-1 cleavage mutants samples were independently prepared and imaged more than once except for the MA-NC mutant which was prepared and imaged once. All replicates gave consistent results. For each sample, thousands of viruses were imaged.<br>2D crystallography: Grids were prepared in triplicate, and the results of all three replicates are included in the presented data.<br>Virus fusion experiment: Plotted points are from three biological replicates with three technical replicate per biological replicate. Each biological replicate represents one aliquot from a bulk preparation of viral particles.
Randomization	cryo-EM data: all datasets were randomly split to two independent halves and processed separately according to the "gold standard" procedure.<br>2D crystallography: Imaging locations within one grid square were chosen randomly. Subsequent analysis does not involve allocation to experimental groups<br>Virus fusion experiment: NA (experiment does not involve allocation to experimental groups)

Blinding | No blinding was performed.

Reporting for specific materials, systems and methods

We require information from authors about some types of materials, experimental systems and methods used in many studies. Here, indicate whether each material, system or method listed is relevant to your study. If you are not sure if a list item applies to your research, read the appropriate section before selecting a response.

Materials & experimental systems

n/a	Involved in the study
☐	☒ Antibodies
☐	☒ Eukaryotic cell lines
☒	☐ Palaeontology and archaeology
☒	☐ Animals and other organisms
☒	☐ Clinical data
☒	☐ Dual use research of concern
☒	☐ Plants

Methods

n/a	Involved in the study
☒	☐ ChIP-seq
☒	☐ Flow cytometry
☒	☐ MRI-based neuroimaging

Antibodies

Antibodies used

sheep αCA, polyclonal, 1:5,000 [in-house];
rabbit αNC, polyclonal, 1:400 [in-house];
Donkey anti-sheep IgG (H&L) Antibody DyLight™ 800 Conjugated, 1:10,000 [Rockland Cat# 613-731-168, RRID:AB_220181];
and Donkey anti-rabbit IgG IRDye 680 Conjugated 1:10,000 [LI-COR Biosciences Cat# 926-32223, RRID:AB_621845].

Validation

Antibodies were used for Western blot only.
sheep αCA, polyclonal. The antiserum has been raised against purified recombinant HIV-1 CA. It was used for immunoblotting in previous studies (e.g., DOI: 10.1074/jbc.M109.027144 ; DOI: 10.1128/JVI.01704-12; DOI: 10.1128/JVI.00750-15), yielding the expected band patterns.
Rabbit αNC, polyclonal. The antiserum has been raised against purified recombinant HIV-1 NC. It was used for immunoblotting in previous studies (e.g., DOI: 10.1128/JVI.01704-12), yielding the expected band patterns.

Eukaryotic cell lines

Policy information about cell lines and Sex and Gender in Research

Cell line source(s) | HEK293T (RRID:CVCL_0063), HEK293 ATCC number CRL-1573.

Authentication | HEK293T Genetic characteristics were confirmed by PCR-single-locus-technology (Eurofins; Promega power Plex 21 PCR Kit). HEK293 cells were authenticated by STR profiling.

Mycoplasma contamination | HEK293T and HEK293 cells were regularly tested for mycoplasma contamination and tested negative.

Commonly misidentified lines (See ICLAC register) | No commonly misidentified cell lines were used in this study.

Plants

Seed stocks

Report on the source of all seed stocks or other plant material used. If applicable, state the seed stock centre and catalogue number. If plant specimens were collected from the field, describe the collection location, date and sampling procedures.

Novel plant genotypes

Describe the methods by which all novel plant genotypes were produced. This includes those generated by transgenic approaches, gene editing, chemical/radiation-based mutagenesis and hybridization. For transgenic lines, describe the transformation method, the number of independent lines analyzed and the generation upon which experiments were performed. For gene-edited lines, describe the editor used, the endogenous sequence targeted for editing, the targeting guide RNA sequence (if applicable) and how the editor was applied.

Authentication

Describe any authentication procedures for each seed stock used or novel genotype generated. Describe any experiments used to assess the effect of a mutation and, where applicable, how potential secondary effects (e.g. second site T-DNA insertions, mosiacism, off-target gene editing) were examined.
